# Is pig organ xenotransplantation era approaching?

**DOI:** 10.1093/lifemedi/lnac032

**Published:** 2022-08-19

**Authors:** Zheng Hu, Minghui Fang, Fei Xu, Kazuhiko Yamada, Yong-Guang Yang

**Affiliations:** Key Laboratory of Organ Regeneration & Transplantation of Ministry of Education, and National-Local Joint Engineering Laboratory of Animal Models for Human Diseases, The First Hospital of Jilin University, Changchun 130061, China; Key Laboratory of Organ Regeneration & Transplantation of Ministry of Education, and National-Local Joint Engineering Laboratory of Animal Models for Human Diseases, The First Hospital of Jilin University, Changchun 130061, China; Key Laboratory of Organ Regeneration & Transplantation of Ministry of Education, and National-Local Joint Engineering Laboratory of Animal Models for Human Diseases, The First Hospital of Jilin University, Changchun 130061, China; Columbia Center for Translational Immunology, Columbia University Medical Center, New York 10032, United States; Key Laboratory of Organ Regeneration & Transplantation of Ministry of Education, and National-Local Joint Engineering Laboratory of Animal Models for Human Diseases, The First Hospital of Jilin University, Changchun 130061, China; International Center of Future Science, Jilin University, Changchun 130012, China

Each year, thousands of patients with end-stage organ failure die worldwide due to shortage of organs for allogeneic organ transplantation. Pig organ xenotransplantation is considered a potential solution to this problem. Robust xenogeneic immune responses impede pig organ survival after transplantation in nonhuman primates (NHPs). Hyperacute rejection (HAR) is a huge challenge in this field [1]. HAR is induced when the carbohydrate xenoantigens are targeted by preformed antibodies. α1,3Gal is one such carbohydrate xenoantigen that is largely expressed on pig cell surface. Generation of α1,3-galactosyltransferase knockout (GTKO) pigs that lack the expression of α1,3Gal, extends pig organ survival time from days to months in NHPs. Incompatibility of immune and coagulation regulatory genes between species also leads to pig organ xenotransplantation failure [1]. The development of novel efficient gene editing (GE) technologies in recent years facilitates the generation of GE pigs to address the challenges mentioned above. There have been reports of pig organ/tissue surviving for years in NHPs after transplantation [2]. Hence, the popularity of pig organ xenotransplantation has increased among researchers and clinicians.

In late 2021, the Alabama [3] and NYU [4] teams independently performed the first and second pig kidney transplantation respectively in brain-dead human subjects. The Alabama team used a 10-GE pigs, in which human complement regulatory genes (hCD55, hCD46), coagulation regulatory genes (hTBM, hEPCR), macrophage function regulatory gene (hCD47), anti-inflammatory gene (hHO1) were incorporated. Three enzyme synthesis genes (GGTA1, CMAH, β4GalNT2) responsible for natural carbohydrate xenoantigen expression and one organ size control gene (GHR) were eliminated, in the organ donor. Two porcine kidneys were bilaterally transplanted in a human subject after native kidney nephrectomies and monitored for 74 h. There was no HAR detected in either porcine kidney during monitoring. There were no human IgM, IgG, C4d complement deposition or cellular immune responses found in the biopsy or explanted samples. However, thrombotic microangiopathy (TMA) with diffuse glomerular capillary congestion and endothelial cell swelling was observed in kidney biopsy from day 1. On day 3, progressive tubular injury with extensive acute tubular necrosis was observed. The right porcine kidney produced urine once after transplantation, whereas urine production of the left porcine kidney was sluggish. The authors suspect that this might be caused by a relatively long warm ischemia time. Neither kidney excreted significant creatinine into the urine and the levels of creatine and BUN (blood urea nitrogen) kept increasing after transplantation. The NYU team transplanted two porcine thymokidneys, in which the thymic tissue was preimplanted under renal capsule 2 months ahead [5], from GTKO pig into two brain-dead patients by anastomosing the xenograft to the femoral artery and vein without native kidney nephrectomy. The porcine kidney was left outside the body on the top of the thigh and monitored for 54 h. There was no HAR, TMA, or deposition of human C4d and IgG observed in either porcine kidneys. However slight focal binding of human IgM on one of the organ samples was observed. Histological and electronic microscopy examination revealed a normal kidney micro-structure without humoral or cell-mediated inflammation. Both porcine kidneys produced urine and performed creatine clearance. Porcine endogenous retrovirus (PERV) was not detected in the three recipients, confirming the consensus that the risk of PERV transmission in humans is very low. Collectively, these two reports confirmed that the elimination of α1,3Gal is effective in preventing HAR occurrence. The native human kidneys might improve porcine kidney survival by reducing the physiological burden of the porcine kidneys. Due to the short time window and limited cases, additional clinical trials are required to address the concerns whether GE pig kidney can support human life in long-term without acute or chronic rejection, and if deletion of the three carbohydrate antigen knockout is superior to deletion of only α1,3Gal in preventing humoral rejection.

One of the most prominent and historical events in the biomedical field this year is the first case of pig heart xenotransplantation in a patient performed by the Drs Mohiuddin and Griffith team in the medical school of the University of Maryland. A 57-year-old patient named David Bennet, who could not be enrolled on the allogeneic organ waiting list, but got special approval by FDA for pig heart transplantation, received a 10-GE pig heart orthotopic xenotransplantation on 7 January 2022 and survived for 2 months [6]. Pig heart functioned normally without apparent rejection at the first few weeks, however, sudden diastolic thickening and xenograft failure was revealed on day 49, and the life support was withdrawn after an irreversible injury to the xenograft was noted. An edematous pig heart with nearly doubled weight was observed on autopsy. An atypical rejection was claimed as scattered myocyte necrosis, interstitial edema, red blood cell extravasation but not microvascular thrombosis was found in the explanted xenograft. Again, PERV transmission was not found in patient sample. Of note, porcine cytomegalovirus was detected in the patients which is known to have a negative impact on organ xenograft survival in NHPs. However, this test confirms that HAR can be surmounted by usage of GE pigs and demonstrates the application of pig hearts in supporting human life.

Currently, longest survival time of pig orthotic heart and life-supporting kidney transplantation in NHPs has surpassed 264 and 557 days, respectively [7, 8]. However, the long-term (>6 months) success rates of porcine heart and kidney xenotransplantation are still low [9]. Other pig organs, such as liver, lung, and intestine, are rapidly rejected or induce severe complications in NHPs [2]. The characteristics of immune responses in xenogeneic organ transplantation are vastly different from the allogeneic ones. For example, cell-mediated immune rejection is the main cause for middle and long-term renal allograft failure in NHPs. However, humoral immune responses commonly occur in xenograft rejection cases [9]. Therefore, further understanding or identifying the cause of xenograft rejection would contribute to the development of effective methods to improve pig organ survival in NHPs.

Theoretically, xenogeneic immune responses can be largely alleviated through appropriate GE in donor pigs by elimination of immunogenic xenoantigens along with the expression of human immune and physiological regulatory proteins. Development of the GGTA1/CMAH/β4GalNT2 triple knockout pig significantly reduced natural carbohydrate antigen burdens [2]. However, a significant amount of natural xenoantigens still exists [2]. The discovery of these antigens and generation of corresponding GE pigs should further facilitate the progress of xenotransplantation. In addition, preexisting anti-HLA (human leukocyte antigen) antibodies induced by presensitization can cross-react with SLA (swine leukocyte antigen) molecules and induce xenograft rejection. As around 1/6 of patients on the organ waiting list have been highly presensitized, generation of GE pig with SLA elimination or modification may further broaden the range of patients suitable for pig organ transplantation. Xeno-organ survival is also influenced by incompatibilities of immune and physiological regulatory signaling between species. Human complement regulatory genes (CD55, CD46), macrophage function inhibitory gene (CD47), and anticoagulant gene (TBM, EPCR) were commonly selected to incorporate into donor pigs. The principle of GE should not be based on “more is better” ideology, as long-term survival has been achieved from GE pigs with only one to three genes edited in NHPs [8].

Immune-suppressive drugs are needed to control xenogeneic immune responses and ensure xeno-organ survival. Due to different mechanisms between xenogeneic and allogeneic immune responses, traditional human applied immune-suppressive drugs do not work well in the pig-to-NHP organ transplantation model. The inclusion of antibodies to block the CD40/CD154 signal is needed to achieve long-term survival of the porcine organ in NHPs [2]. Unlike immune-suppressive drugs, immune tolerance induction mediates specific elimination or irresponsiveness of donor antigen reactive T and B cells while retaining host immunity for pathogens and cancer. Immune tolerance can be induced by establishment of donor/host mixed bone marrow chimerism and donor thymic tissue implantation [10]. The former approach has been successfully applied in human kidney allogeneic transplantation and small animal models of xenotransplantation. The latter has been shown to markedly promote pig organ survival in NHPs [10]. Successful induction of xenogeneic immune tolerance in humans would further facilitate clinical xeno-organ transplantation.

**Figure 1.**
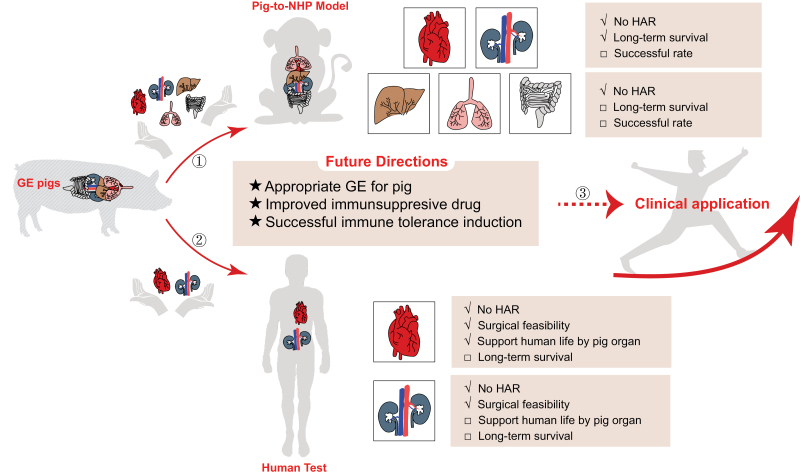
Current status and future direction of pig organ xenotransplantation. ① Great progress and remanent problem in pig-to-NHP organ transplantation. ② Major findings and limitations for recent pig-to-human organ transplantation tests. ③ Future direction of pig organ xenotransplantation investigation and translation.

Great progress has been made in porcine organ xenotransplantation in past few decades (Fig. 1). Considering the fact that allogeneic organ survival in NHPs is always lower than in patients [9], the outcome of pig organ xenotransplantation in humans may possibly be better than in NHP models. Further clinical trials are essential to address the relevant concerns and contribute to the translation of pig organ xenotransplantation in the clinic.
